# Biphasic onset of splenic apoptosis following hemorrhagic shock: critical implications for Bax, Bcl-2, and Mcl-1 proteins

**DOI:** 10.1186/cc6772

**Published:** 2008-01-22

**Authors:** Arwed Hostmann, Kerstin Jasse, Gundula Schulze-Tanzil, Yohan Robinson, Andreas Oberholzer, Wolfgang Ertel, Sven K Tschoeke

**Affiliations:** 1 Institute of Experimental Medicine, Charité – University Medical School Berlin, Campus Benjamin Franklin, Krahmerstraße 6-10, 12207 Berlin, Germany; 2 Department of Biology, Chemistry and Pharmacy, Free University of Berlin, Takustraße 3, 14195 Berlin, Germany; 3 Department of Trauma and Reconstructive Surgery, Charité – University Medical School Berlin, Campus Benjamin Franklin, Hindenburgdamm 30, 12200 Berlin, Germany; 4 Department of Joint and Sport Surgery, Klinik Pyramide am See, Bellerivestraße 34, 8034 Zürich, Switzerland

## Abstract

**Introduction:**

The innate immune response to trauma hemorrhage involves inflammatory mediators, thus promoting cellular dysfunction as well as cell death in diverse tissues. These effects ultimately bear the risk of post-traumatic complications such as organ dysfunction, multiple organ failure, or adult respiratory distress syndrome. In this study, a murine model of resuscitated hemorrhagic shock (HS) was used to determine the apoptosis in spleen as a marker of cellular injury and reduced immune functions.

**Methods:**

Male C57BL-6 mice were subjected to sham operation or resuscitated HS. At t = 0 hours, t = 24 hours, and t = 72 hours, mice were euthanized and the spleens were removed and evaluated for apoptotic changes via DNA fragmentation, caspase activities, and activation of both extrinsic and intrinsic apoptotic pathways. Spleens from untreated mice were used as control samples.

**Results:**

HS was associated with distinct lymphocytopenia as early as t = 0 hours after hemorrhage without regaining baseline levels within the consecutive 72 hours when compared with sham and control groups. A rapid activation of splenic apoptosis in HS mice was observed at t = 0 hours and t = 72 hours after hemorrhage and predominantly confirmed by increased DNA fragmentation, elevated caspase-3/7, caspase-8, and caspase-9 activities, and enhanced expression of intrinsic mitochondrial proteins. Accordingly, mitochondrial pro-apoptotic Bax and anti-apoptotic Bcl-2 proteins were inversely expressed within the 72-hour observation period, thereby supporting significant pro-apoptotic changes. Solely at t = 24 hours, expression of the anti-apoptotic Mcl-1 protein shows a significant increase when compared with sham-operated and control animals. Furthermore, expression of extrinsic death receptors were only slightly increased.

**Conclusion:**

Our data suggest that HS induces apoptotic changes in spleen through a biphasic caspase-dependent mechanism and imply a detrimental imbalance of pro- and anti-apoptotic mitochondrial proteins Bax, Bcl-2, and Mcl-1, thereby promoting post-traumatic immunosuppression.

## Introduction

Hemorrhagic shock (HS) is a commonly encountered complication within a blunt traumatic or surgical injury. The consecutive immune response induces a variety of immune dysfunctions, which promote increased susceptibility to infections and post-traumatic complications like multiple organ dysfunction syndrome, multiple organ failure, or adult respiratory distress syndrome [1-4]. Moreover, it has been reported that trauma hemorrhage or ischemia/reperfusion injury is associated with cell-mediated and parenchymal dysfunctions characterized by the imbalanced production of pro-inflammatory and anti-inflammatory cytokines, reactive oxygen species, and arachidonic acid metabolites [5-12]. There is increasing evidence that HS reduces the proliferative capacity of splenocytes and lymphokine release [13], attenuates macrophage antigen presentation and cytokine release [14], and consecutively impairs humoral immunity [15]. In this regard, recent data evaluating trauma-induced organ dysfunctions have suggested that programmed cell death (apoptosis) plays a critical role in the promotion of post-traumatic complications [16-18]. Therefore, it might be hypothesized that the magnitude of cellular or parenchymal injury after trauma hemorrhage may be attributed, in part, to the rate of apoptosis induced by the respective event. To date, only a few studies following trauma hemorrhage have focused on functional and immunological alterations of the spleen as being one of the most powerful secondary immunological organs [19-22]. Thus, further investigation focusing on splenic immune alteration induced by trauma hemorrhage might help to elucidate the impact of the spleen in the development of post-traumatic immunosuppression.

In physiological states, apoptosis plays an important role in normal development as well as in tissue proliferation. It requires a precise regulation while maintaining the cellular homeostasis [23]. The best-investigated downstream signalling pathways of apoptosis have been described as being predominantly caspase-dependent, following either the extrinsic receptor-mediated activation of caspase-3/7 via binding to members of the tumor necrosis factor receptor (TNFR) superfamily (for example, Fas receptor [CD95] and TNFR-I [CD120α]) or intrinsic mitochondria-induced release of cytochrome c with subsequent activation of caspase-9 and caspase-3, respectively [24]. As the intrinsic pathway is controlled by members of the mitochondrial membrane-bound Bcl-2 family, previous studies on patients with sepsis and shock have demonstrated an essential role of the anti-apoptotic Bcl-2 protein for cell survival [25]. The following murine study focuses on the time-dependent activation of splenic apoptosis via DNA fragmentation, the activation of receptor-mediated extrinsic pathway via the death receptors CD120α and CD95, and the intrinsic mitochondria-related apoptotic pathway by the differential expression of mitochondrial Bax, Bcl-2, and Mcl-1 proteins in regard to the HS-induced risk for post-traumatic immunosuppression.

## Materials and methods

This study was approved by the Institutional Animal Care and Use Committee (LAGetSi, Berlin, Germany). All research was conducted in compliance with the Animal Welfare Act and other federal statues and regulations relating to animals and experiments involving animals.

### Animal preparation and experimental groups

Male C57BL/6 mice between 8 and 12 weeks of age (25 to 30 g) were used in this study. Mice were maintained on a standard 12-hour light cycle and provided with standard rodent chow and water *ad libitum*. Mice were randomly assigned to three groups with six male mice each: control group, sham group, and HS group. HS mice underwent the surgical procedures mentioned below. Sham mice were subjected to the same surgical operations except withdrawing blood and resuscitation. Control mice did not undergo any surgical procedure. All surgical procedures were performed under initial anesthesia with the use of intraperitoneal injection of 120 mg/kg ketamine 10% (WDT, Garbsen, Germany) and 6 mg/kg xylacine (Rompun 2%; Bayer AG, Leverkusen, Germany). An adequate plane of anesthesia was assumed when the animals were unable to right themselves after being placed on their backs as well as when they were unable to respond to toe pinch.

### Hemorrhagic shock model

Animals were anesthetized and placed in a supine position. Groins of both femoral arteries were aseptically cannulated using a microcatheter (Fine Science Tools, Heidelberg, Germany). One catheter was connected to a blood pressure analyzer (Micro-Med, Inc., Louisville, KY, USA) for constant recording of heart rate and mean systolic and diastolic arterial blood pressures. The contralateral catheter was used for withdrawing blood and fluid resuscitation. HS animals were rapidly bled to a mean blood pressure of 35 ± 5 mm Hg (mean blood volume 532 ± 43 μL), which was maintained for 60 minutes. At the end of this period, animals were resuscitated with isotonic 0.9% NaCl solution (3× of the shed blood withdrawn) using a perfusor (B. Braun Medical AG, Sempach, Switzerland) over 30 minutes, following catheter removal, vessel ligation, and closing of the incisions. Hemorrhaged and resuscitated animals were sacrificed on defined time points (immediately after resuscitation [t = 0 hours] as well as at t = 24 hours and t = 72 hours thereafter) by cervical decapitation. The spleen was aseptically removed and administrated for further analysis.

### Cell counting

Lymphocyte cell counting was performed by withdrawing 20 μL of peripheral blood from the caudal tail vein. Cell counts were analyzed in an ABC Animal Blood Counter (scil animal care company, Viernheim, Germany).

### Splenocyte isolation

Spleens were carefully removed in an aseptic manner, washed in Petri dishes containing phosphate-buffered saline (PBS), and placed onto 40-μm nylon-mesh cell strainers (Becton Dickinson, Heidelberg, Germany). A small syringe plunger was used to homogenize spleen tissue through the cell strainer. The remaining cell suspension was washed and resuspended in PBS following cell staining, caspase activity assays, real-time polymerase chain reaction (PCR), and Western blot as described below. Splenic cell suspension was centrifuged at 300 *g *for 5 minutes and washed in buffer containing PBS, 2% fetal calf serum, and Polymyxin B. Cells (0.5 × 10^6^) were resuspended in staining buffer (containing PBS w/o Mg^2+^/Ca^2+^, 1% albumin fraction V, and 0.01% NaN_3_) for further fluorescence activated cell sorting analysis. Additionally, splenic cell suspension was resuspended in RNAlater (Qiagen, Hilden, Germany) for further RNA isolation or in lysis buffer (containing 25 mM HEPES [4-(2-hydroxyethyl)-1-piperazineethanesulfonic acid] [pH 7.5], 0,1% Triton × 100, 5 mM MgCl_2_, 2 mM dithiothreitol [DTT], 1 mM EGTA [ethylene glycol-bis (2-aminoethylether)-N,N,N,N-tetra acetic acid]) containing protein inhibitors (Complete Mini; Roche Diagnostics, Mannheim, Germany) for further Western blot analysis and caspase activity assays, respectively.

### Flow cytometry

Freshly isolated mouse splenocytes were analyzed by direct labeling procedures using primary antibodies anti-mouse CD3 (Invitrogen, Karlsruhe, Germany), anti-mouse CD120α (BioLegend, San Diego, CA, USA), and anti-mouse CD95 (BD Pharmingen, Heidelberg, Germany) and their respective isotype controls. Data acquisition was performed using a FACSCalibur flow cytometer and Cell Quest software (Becton Dickinson). Further data analysis was performed using FlowJo software for PC (TreeStar Inc., Ashland, OR, USA).

### Caspase activity assay

Apoptotic cell death-inducing caspase-3/7, caspase-8, and caspase-9 activity was determined in protein lysates from murine splenocytes. Equal volumes containing 30 μg of protein were applied to the caspase-3/7 activity and caspase-8/-9 activity assays using the Apo-ONE Homogeneous and CaspaseGlo systems (Promega, Mannheim, Germany), respectively. Caspase-3/7 activity was determined via emission intensity of fluorescence (excitation wavelength 490 nm and emission wavelength 535 nm), and caspase-8/-9 activity via emission of luminescence, using a GeniusSpectra Fluorplus fluorescence spectrometer (Tecan Deutschland GmbH, Crailsheim, Germany).

### RNA isolation, cDNA synthesis, and real-time polymerase chain reaction

Total T-cell RNA of murine splenocytes was isolated using an RNeasy Mini Kit (Qiagen) according to the manufacturer's instructions. RNA quantity and quality were evaluated with the RNA 6000 Nano Assay from Agilent Technologies (Waldbronn, Germany). From total RNA, 1 μg was denatured at 75°C for 10 minutes in the presence of oligo-primers (pd(T)12–18) (Amersham Buchler, now part of GE Healthcare, Little Chalfont, Buckinghamshire, UK) and reversely transcribed into cDNA using Molony mouse leukemia virus (Invitrogen) in the presence of frozen storage buffer (Invitrogen), 250 μM dNTPs, 0.01 M DTT, 4 U DNase, and 20 U RNasin (Promega) at 37°C for 30 minutes, followed by heating at 75°C for 5 minutes for DNase activation. After cooling at 4°C, cDNA synthesis was performed at 42°C for 60 minutes. Aliquots (1 μL) of the resulting cDNA were amplified by real-time PCR using a QuanTitect Probe PCR Kit (Qiagen). Primer pairs for Bax and Bcl-2 detection were obtained from the QuanTitect Gene Expression Assay (Qiagen). The primer pair for the β-actin housekeeping gene was used as a reference control (QuanTitect Primers; Qiagen). All assays were performed in an Opticon I Real-Time Cycler from MJ Research (Bio-Rad Laboratories, Inc., Munich, Germany) as follows: primary step of 2 minutes at 50°C, 15 minutes at 95°C, 46 cycles of 15 seconds at 94°C, 30 seconds at 56°C, and 30 seconds at 76°C, according to the manufacturer's protocol.

### DNA fragmentation

The DeadEnd Fluorometric TUNEL (terminal deoxynucleotidyl transferase-mediated dUTP-biotin nick end-labeling) System Kit (Promega Corporation, Madison, WI, USA) on splenic frozen sections was used to detect *in situ *DNA fragmentation. For this purpose, splenic tissues were embedded in Tissue Tec (Sakura, Zoeterwoude, The Netherlands) immediately after removal and frozen in liquid nitrogen. Tissue sections were obtained by cutting 6-μm blocks on a microtome (model RM 2155; Leica, Wetzlar, Germany). Each section was mounted onto a microscope slide and underwent standardized TUNEL staining. The resulting stained sections were examined for apoptotic cells by a fluorescence microscope (Axioskop 40; Carl Zeiss, Jena, Germany) followed by visualization with a C-4000 camera (Olympus, Hamburg, Germany). Quantificational TUNEL analyses were performed by counting the rate of TUNEL-positive cells within a total number of 200 cells using the Alpha Digidoc software (Alpha Innotech, Grödig/Salzburg, Austria).

### Western blot

Protein lysates from isolated splenocytes were thawed on ice. Equal amounts of protein (60 μg) were boiled and denatured in sample buffer at 95°C for 5 minutes and then separated by 12% Tris-glycine SDS-PAGE. Afterward, proteins were transferred to a nitrocellulose membrane by wet blotting. Equal protein loading was examined by Ponceau S staining. Afterward, the membrane was blocked and incubated overnight at 4°C with primary host species rabbit anti-mouse Bax, mouse anti-mouse Bcl-2 (Santa Cruz Biotechnology, Inc., Heidelberg, Germany) (1:300 diluted in PBS, 0.05% Tween 20, and 5% skim milk powder) and rabbit anti-mouse Mcl-1 (BioLegend) (diluted 1:500 in PBS, 0.05% Tween 20, and 3% bovine serum albumin) polyclonal antibodies. Finally, membranes were washed and incubated with the secondary goat anti-rabbit (1:2,500) or goat anti-mouse IgG (1:5,000) horseradish peroxidase-conjugated antibodies (DakoCytomation, Hamburg, Germany) for 2 hours. After washing, detection was performed by non-radioactive chemiluminescence using RotiLumin (Carl Roth, Karlsruhe, Germany) and an LAS 3000 Image Reader (Fujifilm, Düsseldorf, Germany). Antibody against the cytosolic marker β-actin (1:2,500 for 45 minutes) (Sigma-Aldrich, Munich, Germany) was used to re-examine equal sample loading and detection of contamination. Quantificational Western blot analyses were performed using the Alpha Digidoc software.

### Presentation of data and statistics

Results are presented as the mean (± standard error of the mean). Differences between experimental groups were considered significant at a *p *value of less than 0.05 as determined by the analysis of variance (Bonferroni/Dunn) test and the Mann-Whitney test.

## Results

A total of 42 mice were subject to HS or sham operation or were healthy controls. HS treatment led to a rapid decrease of the mean arterial pressure after blood withdrawal from initial values of 97.7 ± 10.3 mm Hg to 35 ± 5 mm Hg (data not shown). The average volume of blood withdrawn comprised 532 ± 43 μL. In sham-operated mice, no significant changes in blood pressure compared with control animals were observed (data not shown).

### Lymphocyte cell counts

**Figure 1:**
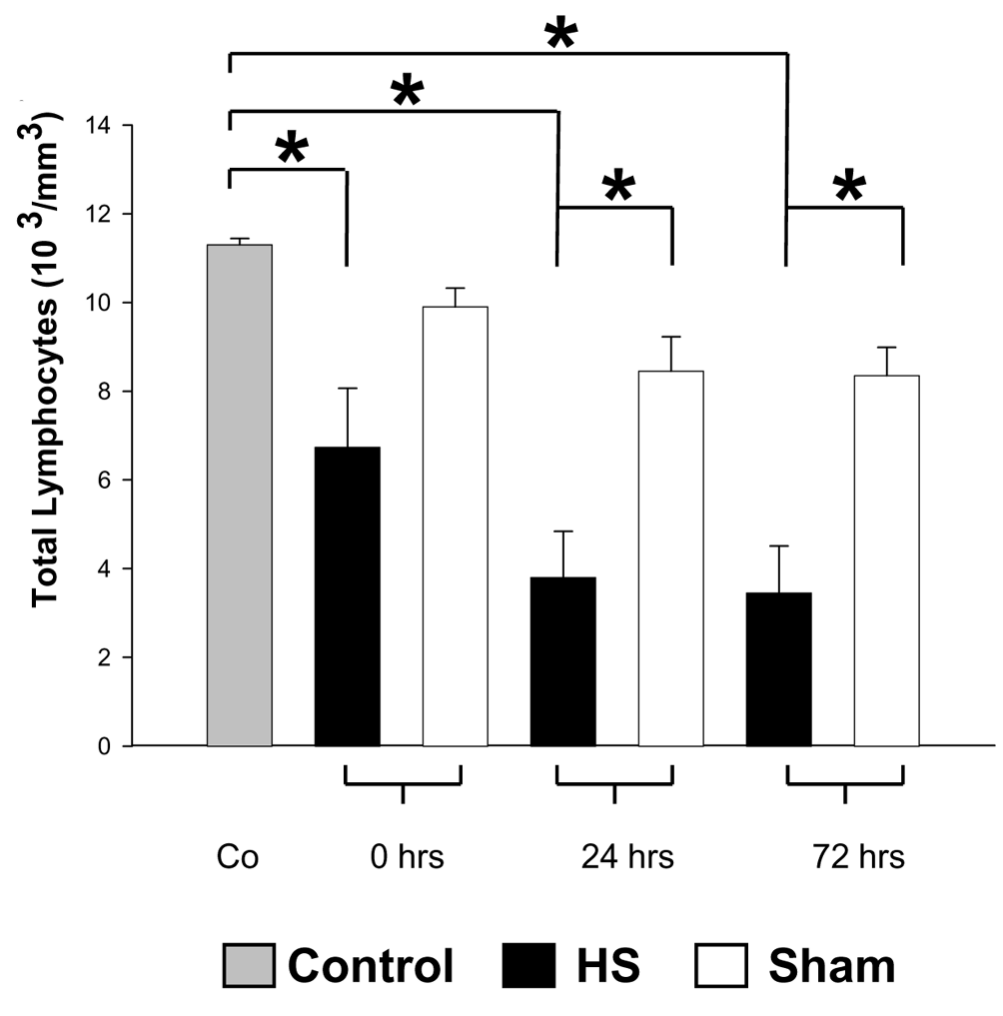
Total lymphocytes after hemorrhagic shock (HS). HS-induced risk for immunosuppression was confirmed by changes of total lymphocytes in murine peripheral blood. Peripheral blood from HS, sham, and control animals was obtained as described in Materials and methods and analyzed by differential hemogram. *P < 0.05 as determined by analysis of variance (with *post hoc *Bonferroni/Dunn) test and Mann-Whitney test.

Peripheral whole blood from control mice was directly obtained by puncture of the caudal vein and immediately processed for further analyses. Blood from animals subjected to HS was obtained and processed in an analogous manner after resuscitation and vessel ligation at t = 0 hours and at t = 24 hours and t = 72 hours after resuscitated hemorrhage. Blood from sham-operated mice was obtained and processed in an analogous manner after removal of the catheter and vessel ligation. Total lymphocyte cell counts revealed a significant lymphocytopenia in mice undergoing HS compared with those of the sham group and control animals (Figure 1). Absolute lymphocyte decrease was observed from time point t = 0 hours onward without regaining baseline levels within the consecutive 72-hour observation period. However, mainly for two reasons, peripheral blood lymphocyte cell counts may not accurately reflect the total number of lymphocytes. First, peripheral blood lymphocytes represent only a small fraction of the total body lymphocytes whereas the majority of lymphocytes are found in lymphoid tissues like lymph nodes, Payer's patch, or spleen. Second, a potential dilutional effect provoked by the resuscitation must be considered.

### Hemorrhagic shock-induced lymphocyte apoptosis and caspase activity

**Figure 2:**
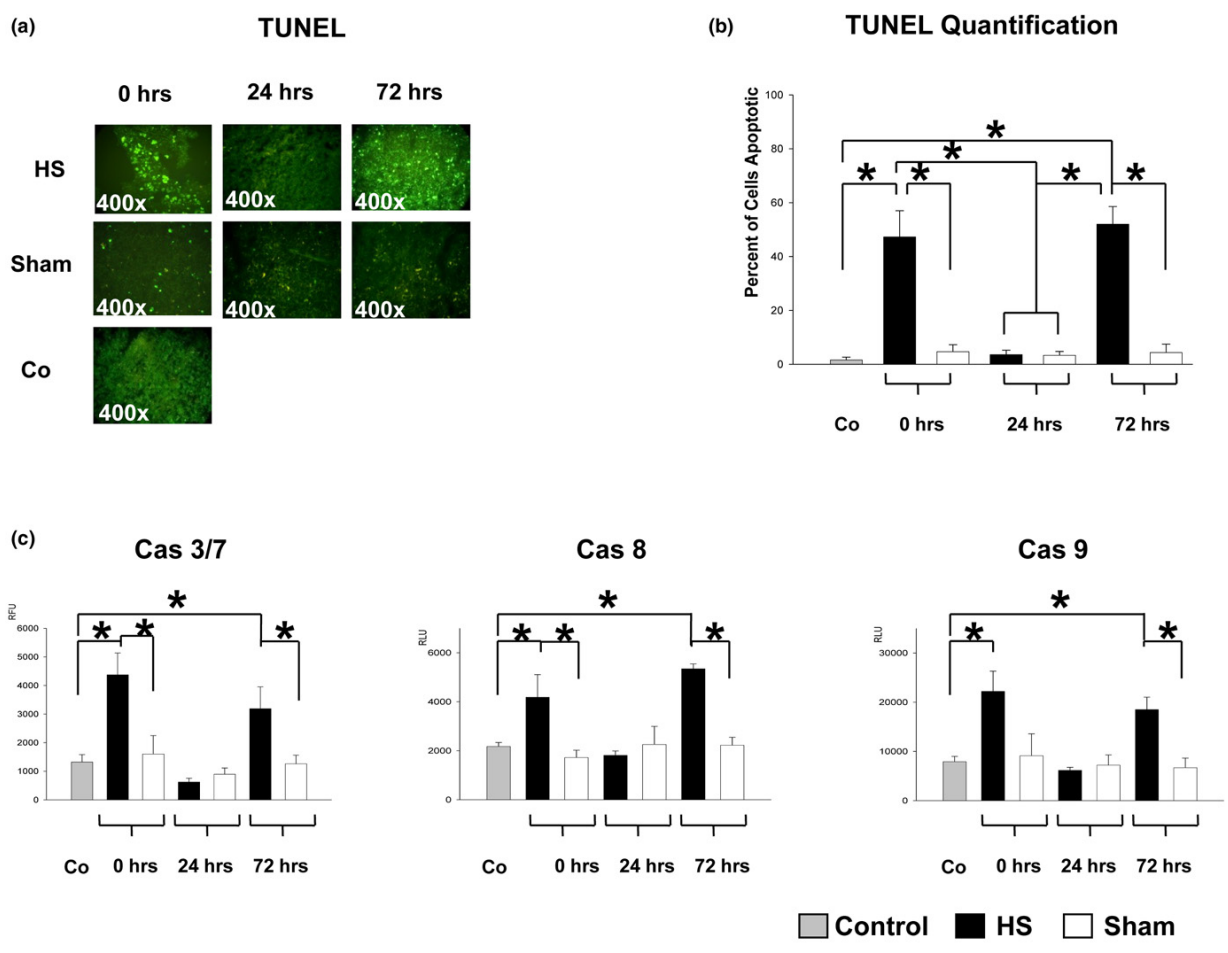
Hemorrhagic shock (HS)-induced apoptosis of murine spleen. **(a) **DNA fragmentation as shown by TUNEL staining. Splenocytes were isolated from HS and sham animals at t = 0 hours, t = 24 hours, and t = 72 hours after hemorrhage as well as from control animals. Results are representative of at least three animals per group and controls. **(b) **Quantificational analysis of DNA fragmentation. Results are representative of at least three animals per group and controls. **(c) **Activity of death-receptor-mediated effector caspase-3/7 and caspase-8 as well as mitochondria-related caspase-9 activity within the entire observation period. *P < 0.05 as determined by analysis of variance (with *post hoc *Bonferroni/Dunn) test and Mann-Whitney test. Co, control; RFU, relative fluorescent units; RLU, relative light units; TUNEL, terminal deoxynucleotidyl transferase-mediated dUTP-biotin nick end-labeling.

Apoptotic lymphocytes in spleen were detected by their fluorescent signal after labelling DNA strand breaks with fluorescein-conjugated nucleotides. Figure 2 shows a representative TUNEL stain (a) and quantificational analysis (b) of freshly isolated and frozen sectioned splenocytes of at least three experiments. In control and sham-operated mice, no or only insular apoptotic cells were observed within the entire observation period (Figure 2a). In resuscitated HS animals, the number of splenocytes showing apoptotic DNA fragmentation was increased at t = 0 hours and t = 72 hours after hemorrhage (Figure 2a). In contrast, 24 hours after HS, most of the splenocytes showed fluorescence signals comparable to those in sham-operated or control mice, demonstrating no observable apoptotic activity (Figure 2a). Accordingly, quantificational analysis of apoptotic DNA fragmentation revealed a significant increase at t = 0 hours and t = 72 hours after hemorrhage, whereas no changes at t = 24 hours occurred, when compared with control and sham animals (Figure 2b). Subsequently, comparative analyses of both receptor- and non-receptor-mediated caspase-3/7 activity in addition to activity of caspase-8 as well as mitochondria-related caspase-9 activity in control, sham-operated, and resuscitated HS mice were performed. Thereby, HS animals demonstrated significantly increased caspase-3/7, caspase-8, and caspase-9 activities at t = 0 hours and t = 72 hours in splenic tissue when compared with the appropriate sham-operated or control animals (Figure 2c). On the other hand, at t = 24 hours after hemorrhage, baseline levels of caspase activities were monitored (Figure 2c).

### Hemorrhagic shock-induced death receptor expression

**Figure 3:**
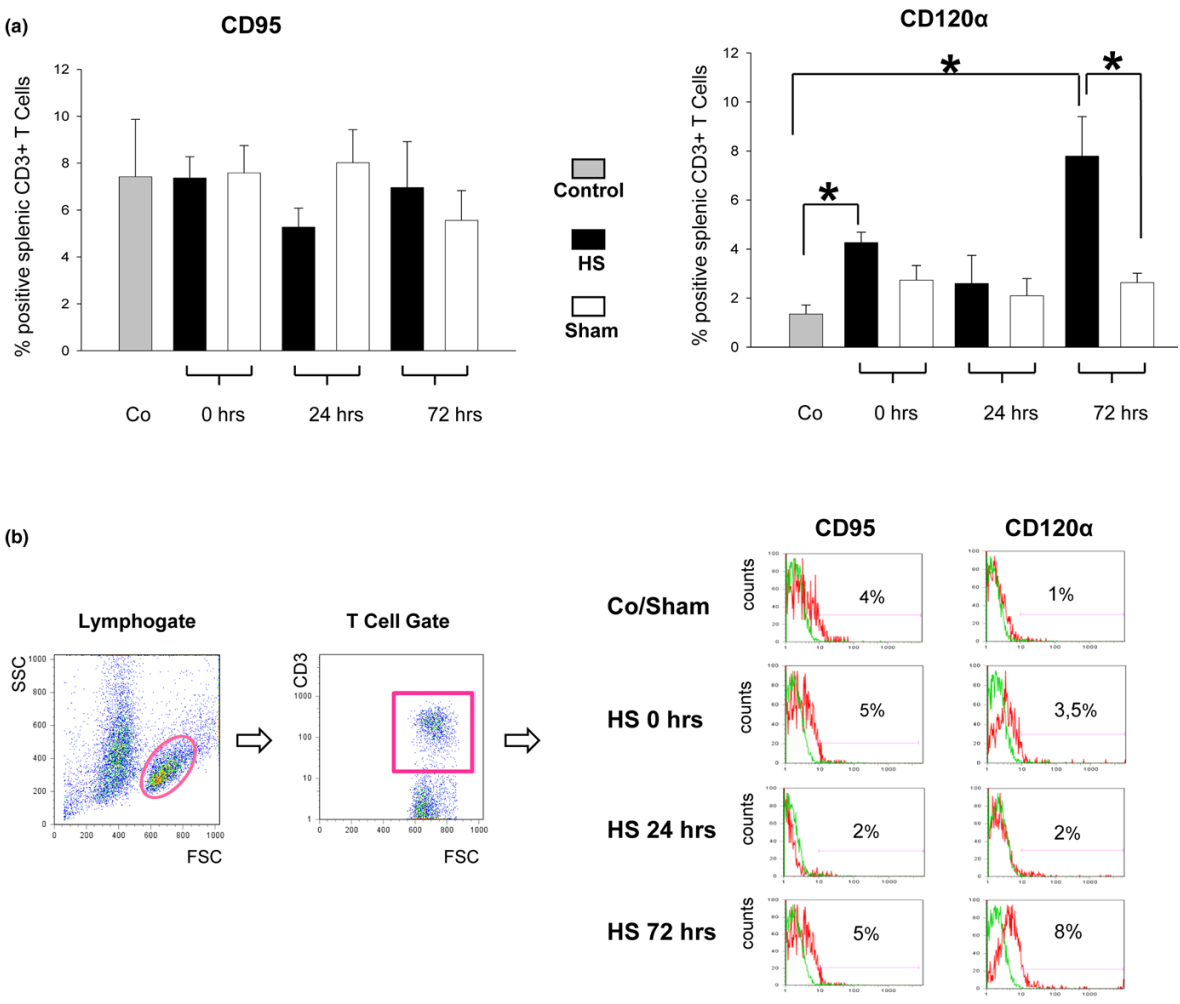
Expression of death receptors after hemorrhagic shock (HS). **(a) **HS-induced expression of extrinsic CD95 and CD120α death receptors in murine spleen. **(b) **Gating strategy and percentage of splenic CD3 T cells positive for CD95 as well as for CD120α compared with healthy controls and sham-operated animals (representative dot plots and histograms for at least three experiments, green line = isotype control, red line = specific marker). *P < 0.05 as determined by analysis of variance (with *post hoc *Bonferroni/Dunn) test and Mann-Whitney test. Co, control; FSC, forward scatter; SSC, side scatter.

Splenic death receptor CD95 and CD120α protein expression in control, sham-operated, and HS animals was examined by flow cytometry. Previous studies have shown that CD95 is expressed by the majority of immature T cells in the normal mouse thymus, but to a lower extent in normal splenic lymphocytes [26-28]. In this study, splenic CD95 protein expression of control animals did not differ significantly within the entire observation period when compared with sham- and HS-operated mice (Figure 3a,b). In contrast, CD120α was upregulated at t = 0 hours and t = 72 hours in HS animals (Figure 3a,b). Twenty-four hours after hemorrhage, the level of CD120α expression was rather comparable to those of sham-operated mice and control animals. However, CD120α expression was consistent with appropriate results of caspase-3/7 and caspase-8 activities at t = 0 hours, t = 24 hours, and t = 72 hours after hemorrhage (Figure 2c). Therefore, a contribution of the CD120α-mediated pathway to splenic apoptosis cannot be excluded but might play a minor role.

### Hemorrhagic shock-induced mitochondria related pro- and anti-apoptotic proteins

**Figure 4:**
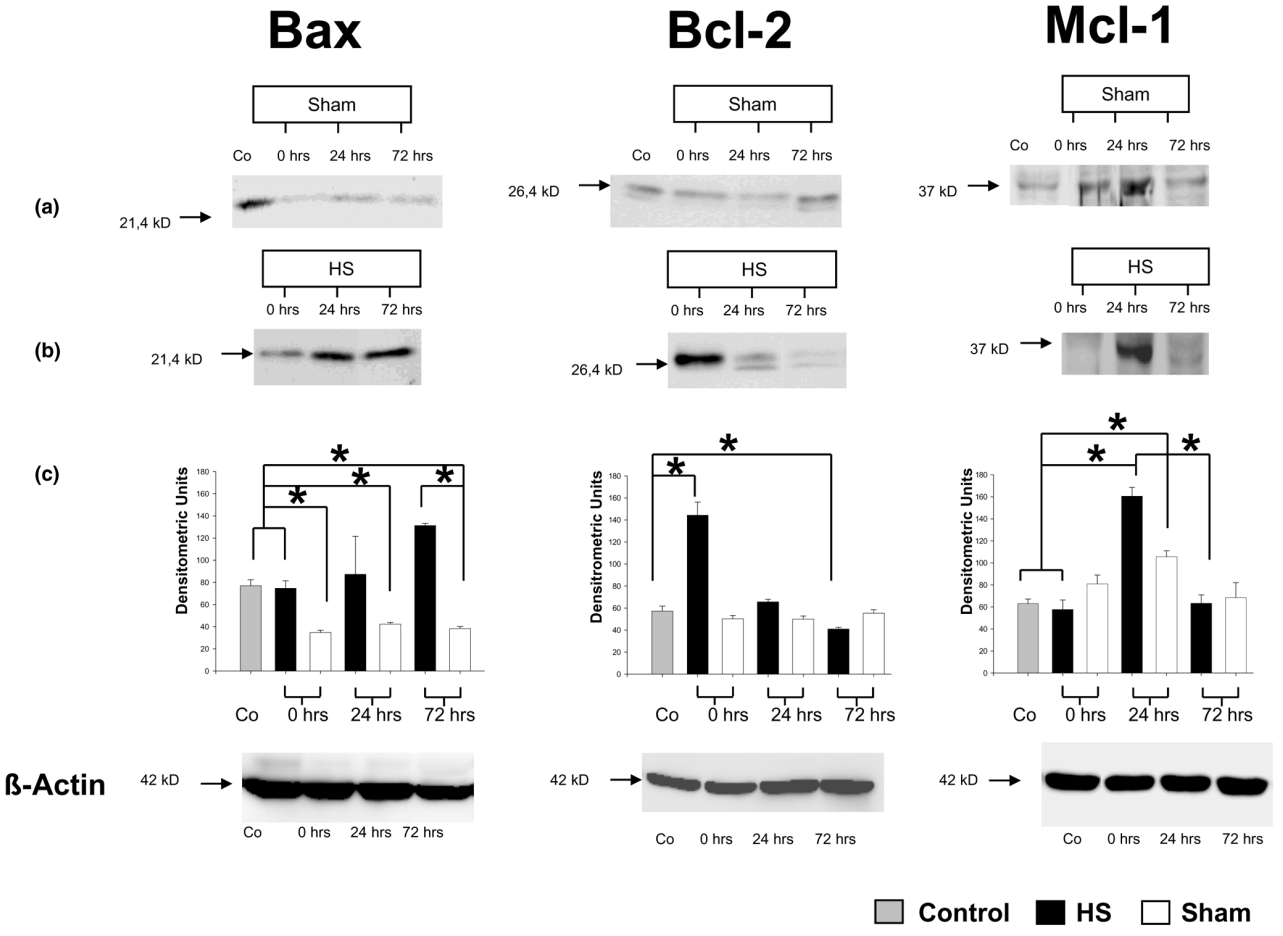
Protein expression of mitochondrial proteins after hemorrhagic shock (HS). Western blot analysis of splenic Bax (left), Bcl-2 (middle), and Mcl-1 (right) expression compared to the housekeeping gene β-actin (lower part), detected in splenocytes of sham and controls (a) as well as in HS animals (b). Results are representative of at least three animals per group and controls. *P < 0.05 as determined by analysis of variance (with *post hoc *Bonferroni/Dunn) test and Mann-Whitney test. Co, control.

To prove the involvement of mitochondria-related proteins in the downstream apoptotic signalling cascade in spleen after HS, we investigated the protein expression of pro-apoptotic Bax as well as anti-apoptotic Bcl-2 and Mcl-1 by semi-quantitative Western blot analysis. Figure 4 demonstrates a representative Western blot of Bax, Bcl-2, and Mcl-1 proteins of at least three experiments. In regard to Bax protein expression, weak expression signals were detected in sham animals within the observed time point whereas control animals showed a higher expression level (Figure 4a, left). Protein expression levels of Bcl-2 in both control animals and animals that underwent a sham procedure did not show any significant differences throughout the entire observation period (Figure 4a, middle). In bright contrast, splenocytes of HS mice showed remarkably divergent values in their Bax and Bcl-2 protein expression levels at t = 0 hours, t = 24 hours, and t = 72 hours after hemorrhage (Figure 4b, left and middle). Moreover, the expression levels of both proteins appeared to be inversely expressed. Throughout the three observation time points, Bcl-2 expression levels were continuously decreasing from t = 0 hours onward, whereas Bax expression was significantly elevated at t = 24 hours and t = 72 hours when compared with t = 0 hours after hemorrhage, indicating a distinct pro-apoptotic shift (Figure 4c, left and middle). Furthermore, protein data of inversely expressed Bax and Bcl-2 protein expression were confirmed by additional analysis of Bax and Bcl-2 mRNA expression using real-time PCR (data not shown).

To elucidate the involvement of additional mitochondrial proteins in splenocyte apoptosis, we sought to introduce another member of the mitochondria-related anti-apoptotic Bcl-2 family, Mcl-1 (Figure 4a–c, right). However, consistent expression levels of the Mcl-1 protein were detectable in sham-operated and control animals, except for an increased expression at t = 24 hours after hemorrhage. In contrast, Mcl-1 expression in splenocytes of HS-treated animals was significantly enhanced at t = 24 hours after hemorrhage when compared with t = 0 hours and t = 72 hours, supporting our hypothesis that splenic cells are rescued from apoptosis by potential involvement of Mcl-1 (Figure 4b,c, right). Additionally, the time course of anti-apoptotic Mcl-1 expression is in line with the appropriate caspase-3/7 and caspase-9 activities and correlates with the HS-induced DNA fragmentation demonstrated in Figure 2.

## Discussion

Previous human studies have shown that serious injury induces a variety of both morphological and functional changes in lymphocytes, which are indicated to play a major part in post-traumatic immunosuppression [29-32]. To date, reports on animal models and clinical studies have been able to demonstrate that the consecutive dysfunction of key immune effector cells after trauma hemorrhage and shock, such as lymphocytes, may be associated with induced and prematurely activated apoptosis [33-36]. Animal studies have clearly shown that hemorrhage alone is sufficient to cause a variety of parenchymal alterations, including cellular damage and cytotoxic effects [37-39]. Additionally, hemorrhage alone has been shown to induce thymocyte apoptosis, which is assumed to contribute to the deregulation of immune responses and the development of post-traumatic immunosuppression [18]. Furthermore, the spleen as an important homing of T lymphocytes appears to be involved in the immune response following hemorrhage in combination with trauma [19]. After ischemia/reperfusion injury, apoptosis also can be induced in liver [6], kidney [40], heart [41], and brain [42]. On the basis of our experimental settings presented in the following study, previous data have critically discussed the type fluid resuscitation following HS in regard to the extent of parenchymal apoptosis. For example, an increased hepatic, intestinal, and pulmonary apoptotic activity has been reported during resuscitation with lactated Ringer solution [43,44]. In contrast, hypertonic saline infusion exhibits protective properties reducing cellular apoptosis, tissue damage, and susceptibility to sepsis [45-48]. Nevertheless, our correlating results obtained by TUNEL stain and caspase activity assays allowed us to propose that HS induces both pro- and anti-apoptotic changes in murine splenocytes. Biphasic activation of parenchymal apoptotic processes in various animal models of injury has been reported by the use of several pharmacological substances (for example, staurosporin [49] or bleomycin [50]).

In the present study, apoptotic changes of murine splenocytes from animals subjected to HS and resuscitation were compared with sham-operated and control animals and investigated within an observation period of 72 hours. Our results demonstrate a time-dependent and biphasic activity (at t = 0 hours and t = 72 hours after hemorrhage) of key apoptosis-inducing enzymes (caspase-3/7 and caspase-8) and apoptosis-related CD120α expression along with corresponding apoptotic DNA fragmentation via TUNEL analysis. However, the minor expression levels of both CD120α and CD95 death receptors do not necessarily mean that the receptor-mediated pathway is not activated, since it requires not only the receptors but also binding of their ligands. Despite this, TUNEL staining analysis and confirmative results of increased caspase-3/7 and caspse-8 activity of splenocytes over the 3-day observation period support the common notion that the early splenocyte apoptosis is associated, at least in part, with caspase-dependent and both intrinsic and extrinsic apoptotic signalling pathways. Our analyses of HS-induced apoptotic changes in splenocytes indicated a biphasic activation of caspase-9 with a corresponding increase of pro-apoptotic Bax and repressed anti-apoptotic Bcl-2 protein expression. This is partially in line with recent observations showing pulmonary upregulation of Bax protein expression in rats following lactated Ringer solution and hetastarch resuscitated HS [44]. Remarkably, our results suggest a downregulated splenocyte apoptosis at t = 24 hours after HS predominantly correlating with an upregulation of the specific anti-apoptotic mediators, namely Mcl-1 as a member of the Bcl-2 family. The essential role of Mcl-1 in regulating cell viability has been established in various experimental settings [51,52], including a murine host-mediated macrophage apoptosis model during pneumococcal infection [53]. In the latter model, a tightly regulated biphasic pattern of macrophage susceptibility to apoptosis has been proposed for optimal killing of bacteria during infection. Following this, a biphasic course of apoptosis regulation seems to be a beneficial feature providing effective host response against serious injury or pathogens. Furthermore, our results suggest the potential involvement of Mcl-1 protein in a possible counter-regulatory mechanism at t = 24 hours after HS. Thus, our results provide evidence of early splenocyte apoptosis triggered by HS, implicating an initial pro-apoptotic shift, counter-regulation, and subsequent rebound effect. It might be proposed that the predominant and specific upregulation of the Mcl-1 protein at t = 24 hours within the early phase of post-hemorrhage recovery contributes to a physiological attempt, thus acting against the potential risk of immunosuppression following HS.

## Conclusion

Our findings demonstrate that HS is sufficient to induce murine splenocyte apoptosis and further itemize time-dependent downstream signalling events within the complex pathophysiology of HS-induced immune alterations, which have not been described in these settings to date. We further provide evidence that splenocyte apoptosis after HS displays a biphasic pattern within the early recovery phase, which is caspase-3/7 – as well as caspase-9-dependent and appears to be mainly intrinsic-mediated via the mitochondrial proteins Bax, Bcl-2, and Mcl-1. Due to the observed activation of caspase-8, an involvement of receptor-mediated pathways in HS-induced apoptotic changes cannot be excluded. In summary, it might be suggested that HS-induced apoptosis-related malfunction of murine splenocytes may be critically involved in the post-traumatic immunosuppression.

## Key messages

• Animal models help to elucidate the complex pathophysiology of trauma-hemorrhage-induced immune responses.

• Immunsuppression following trauma hemorrhage might be caused by parenchymal alterations (for example, in the spleen).

• Murine hemorrhagic shock leads to time-dependent activation of splenic apoptosis

• The biphasic course of apoptosis after hemorrhagic shock displays a biphasic pattern in the early recovery phase in which the intrinsic mitochondria-related Bax, Bcl-2, and Mcl-1 proteins are critically involved.

## Abbreviations

DTT = dithiothreitol; HS = hemorrhagic shock; PBS = phosphate-buffered saline; PCR = polymerase chain reaction; TNFR = tumor necrosis factor receptor; TUNEL = terminal deoxynucleotidyl transferase-mediated dUTP-biotin nick end-labeling.

## Competing interests

The authors declare that they have no competing interests.

## Authors' contributions

AH, in part with AO, drafted the study, carried out all surgical animal procedures, and wrote the manuscript. KJ carried out all described methods. GS-T revised the manuscript and contributed to Western blot analysis and TUNEL stains. YR and WE each contributed substantially to the revision of the manuscript. AO, in part with AH, drafted the study and contributed substantially to the revision of the manuscript. SKT performed statistical analysis and participated in writing the manuscript. All authors have read and approved the final manuscript.

## References

[B1] Chaudry IH, Ayala A (1993). Mechanism of increased susceptibility to infection following hemorrhage. Am J Surg.

[B2] Stephan RN, Kupper TS, Geha AS, Baue AE, Chaudry IH (1987). Hemorrhage without tissue trauma produces immunosuppression and enhances susceptibility to sepsis. Arch Surg.

[B3] Livingston DH, Malangoni MA (1989). An experimental study of susceptibility to infection after hemorrhagic shock. Surg Gynecol Obstet.

[B4] Carrico CJ, Meakins JL, Marshall JC, Fry D, Maier RV (1986). Multiple-organ-failure syndrome. Arch Surg.

[B5] Sasaki H, Matsuno T, Nakagawa K, Tanaka N (1997). Induction of apoptosis during the early phase of reperfusion after rat liver ischemia. Acta Med Okayama.

[B6] Sasaki H, Matsuno T, Tanaka N, Orita K (1996). Activation of apoptosis during the reperfusion phase after rat liver ischemia. Transplant Proc.

[B7] Shah KA, Shurey S, Green CJ (1997). Characterization of apoptosis in intestinal ischaemia-reperfusion injury – a light and electron microscopic study. Int J Exp Pathol.

[B8] Chen J, Jin K, Chen M, Pei W, Kawaguchi K, Greenberg DA, Simon RP (1997). Early detection of DNA strand breaks in the brain after transient focal ischemia: implications for the role of DNA damage in apoptosis and neuronal cell death. J Neurochem.

[B9] Gottlieb RA, Burleson KO, Kloner RA, Babior BM, Engler RL (1994). Reperfusion injury induces apoptosis in rabbit cardiomyocytes. J Clin Invest.

[B10] Fujimoto K, Hosotani R, Wada M, Lee J, Koshiba T, Miyamoto Y, Doi R, Imamura M (1997). Ischemia-reperfusion injury on the pancreas in rats: identification of acinar cell apoptosis. J Surg Res.

[B11] Waxman K (1996). Shock: ischemia, reperfusion, and inflammation. New Horiz.

[B12] Waxman K (1996). What mediates tissue injury after shock?. New Horiz.

[B13] Chaudry IH, Ayala A, Ertel W, Stephan RN (1990). Hemorrhage and resuscitation: immunological aspects. Am J Physiol.

[B14] Ayala A, Perrin MM, Chaudry IH (1990). Defective macrophage antigen presentation following haemorrhage is associated with the loss of MHC class II (Ia) antigens. Immunology.

[B15] Abraham E, Freitas AA (1989). Hemorrhage in mice induces alterations in immunoglobulin-secreting B cells. Crit Care Med.

[B16] Liaudet L, Soriano FG, Szabo E, Virag L, Mabley JG, Salzman AL, Szabo C (2000). Protection against hemorrhagic shock in mice genetically deficient in poly(ADP-ribose)polymerase. Proc Natl Acad Sci USA.

[B17] Xu YX, Wichmann MW, Ayala A, Cioffi WG, Chaudry IH (1997). Trauma-hemorrhage induces increased thymic apoptosis while decreasing IL-3 release and increasing GM-CSF. J Surg Res.

[B18] Xu YX, Ayala A, Monfils B, Cioffi WG, Chaudry IH (1997). Mechanism of intestinal mucosal immune dysfunction following trauma-hemorrhage: increased apoptosis associated with elevated Fas expression in Peyer's patches. J Surg Res.

[B19] Kawasaki T, Fujimi S, Lederer JA, Hubbard WJ, Choudhry MA, Schwacha MG, Bland KI, Chaudry IH (2006). Trauma-hemorrhage induces depressed splenic dendritic cell functions in mice. J Immunol.

[B20] Kawasaki T, Choudhry MA, Schwacha MG, Bland KI, Chaudry IH (2006). Lidocaine depresses splenocyte immune functions following trauma-hemorrhage in mice. Am J Physiol Cell Physiol.

[B21] Oberbeck R, Nickel E, von Griensven M, Tschernig T, Wittwer T, Schmitz D, Pape HC (2002). The effect of dehydroepiandrosterone on hemorrhage-induced suppression of cellular immune function. Intensive Care Med.

[B22] Meldrum DR, Ayala A, Perrin MM, Ertel W, Chaudry IH (1991). Diltiazem restores IL-2, IL-3, IL-6, and IFN-gamma synthesis and decreases host susceptibility to sepsis following hemorrhage. J Surg Res.

[B23] Zimmermann KC, Green DR (2001). How cells die: apoptosis pathways. J Allergy Clin Immunol.

[B24] Oberholzer C, Oberholzer A, Clare-Salzler M, Moldawer LL (2001). Apoptosis in sepsis: a new target for therapeutic exploration. FASEB J.

[B25] Hotchkiss RS, Osmon SB, Chang KC, Wagner TH, Coopersmith CM, Karl IE (2005). Accelerated lymphocyte death in sepsis occurs by both the death receptor and mitochondrial pathways. J Immunol.

[B26] Andjelic S, Drappa J, Lacy E, Elkon KB, Nikolic-Zugic J (1994). The onset of Fas expression parallels the acquisition of CD8 and CD4 in fetal and adult alpha beta thymocytes. Int Immunol.

[B27] Drappa J, Brot N, Elkon KB (1993). The Fas protein is expressed at high levels on CD4+CD8+ thymocytes and activated mature lymphocytes in normal mice but not in the lupus-prone strain, MRL lpr/lpr. Proc Natl Acad Sci USA.

[B28] Ogasawara J, Suda T, Nagata S (1995). Selective apoptosis of CD4+CD8+ thymocytes by the anti-Fas antibody. J Exp Med.

[B29] Hotchkiss RS, Schmieg RE, Swanson PE, Freeman BD, Tinsley KW, Cobb JP, Karl IE, Buchman TG (2000). Rapid onset of intestinal epithelial and lymphocyte apoptotic cell death in patients with trauma and shock. Crit Care Med.

[B30] Walsh DS, Siritongtaworn P, Pattanapanyasat K, Thavichaigarn P, Kongcharoen P, Jiarakul N, Tongtawe P, Yongvanitchit K, Komoltri C, Dheeradhada C, Pearce FC, Wiesmann WP, Webster HK (2000). Lymphocyte activation after non-thermal trauma. Br J Surg.

[B31] Kelly JL, O'Suilleabhain CB, Soberg CC, Mannick JA, Lederer JA (1999). Severe injury triggers antigen-specific T-helper cell dysfunction. Shock.

[B32] Feeney C, Bryzman S, Kong L, Brazil H, Deutsch R, Fritz LC (1995). T-lymphocyte subsets in acute illness. Crit Care Med.

[B33] Guan J, Jin DD, Jin LJ, Lu Q (2002). Apoptosis in organs of rats in early stage after polytrauma combined with shock. J Trauma.

[B34] Pellegrini JD, De AK, Kodys K, Puyana JC, Furse RK, Miller-Graziano C (2000). Relationships between T lymphocyte apoptosis and anergy following trauma. J Surg Res.

[B35] Yamada R, Tsuchida S, Hara Y, Tagawa M, Ogawa R (2002). Apoptotic lymphocytes induced by surgical trauma in dogs. J Anesth.

[B36] Takabayashi A, Kanai M, Kawai Y, Iwata S, Sasada T, Obama K, Taki Y (2003). Change in mitochondrial membrane potential in peripheral blood lymphocytes, especially in natural killer cells, is a possible marker for surgical stress on the immune system. World J Surg.

[B37] Zuckerbraun BS, McCloskey CA, Gallo D, Liu F, Ifedigbo E, Otterbein LE, Billiar TR (2005). Carbon monoxide prevents multiple organ injury in a model of hemorrhagic shock and resuscitation. Shock.

[B38] Zellweger R, Ayala A, Schmand JF, Morrison MH, Chaudry IH (1995). PAF-antagonist administration after hemorrhage-resuscitation prevents splenocyte immunodepression. J Surg Res.

[B39] Zhu XL, Zellweger R, Zhu XH, Ayala A, Chaudry IH (1995). Cytokine gene expression in splenic macrophages and Kupffer cells following haemorrhage. Cytokine.

[B40] Takeda T (1996). [A pathomorphological study on damage and repair process of tubuli after renal ischemia]. Nippon Jinzo Gakkai Shi.

[B41] Fliss H, Gattinger D (1996). Apoptosis in ischemic and reperfused rat myocardium. Circ Res.

[B42] Du C, Hu R, Csernansky CA, Hsu CY, Choi DW (1996). Very delayed infarction after mild focal cerebral ischemia: a role for apoptosis?. J Cereb Blood Flow Metab.

[B43] Deb S, Martin B, Sun L, Ruff P, Burris D, Rich N, DeBreux S, Austin B, Rhee P (1999). Resuscitation with lactated Ringer's solution in rats with hemorrhagic shock induces immediate apoptosis. J Trauma.

[B44] Deb S, Sun L, Martin B, Talens E, Burris D, Kaufmann C, Rich N, Rhee P (2000). Lactated ringer's solution and hetastarch but not plasma resuscitation after rat hemorrhagic shock is associated with immediate lung apoptosis by the up-regulation of the Bax protein. J Trauma.

[B45] Coimbra R, Hoyt DB, Junger WG, Angle N, Wolf P, Loomis W, Evers MF (1997). Hypertonic saline resuscitation decreases susceptibility to sepsis after hemorrhagic shock. J Trauma.

[B46] Powers KA, Zurawska J, Szaszi K, Khadaroo RG, Kapus A, Rotstein OD (2005). Hypertonic resuscitation of hemorrhagic shock prevents alveolar macrophage activation by preventing systemic oxidative stress due to gut ischemia/reperfusion. Surgery.

[B47] Murao Y, Hata M, Ohnishi K, Okuchi K, Nakajima Y, Hiasa Y, Junger WG, Hoyt DB, Ohnishi T (2003). Hypertonic saline resuscitation reduces apoptosis and tissue damage of the small intestine in a mouse model of hemorrhagic shock. Shock.

[B48] Coimbra R, Junger WG, Hoyt DB, Liu FC, Loomis WH, Evers MF (1996). Hypertonic saline resuscitation restores hemorrhage-induced immunosuppression by decreasing prostaglandin E2 and interleukin-4 production. J Surg Res.

[B49] Suzuki K, Azuma Y, Onishi Y, Kizaki H, Ishimura Y (1995). Biphasic effect of staurosporine on thymocyte apoptosis. Biochem Mol Biol Int.

[B50] Koshika T, Hirayama Y, Ohkubo Y, Mutoh S, Ishizaka A (2005). Tacrolimus (FK506) has protective actions against murine bleomycin-induced acute lung injuries. Eur J Pharmacol.

[B51] Smolewska E, Stanczyk J, Robak T, Smolewski P (2006). Inhibited apoptosis of synovial fluid lymphocytes in children with juvenile idiopathic arthritis is associated with increased expression of myeloid cell leukemia 1 and XIAP proteins. J Rheumatol.

[B52] Takagi Y, Du J, Ma XY, Nakashima I, Nagase F (2004). Phorbol 12-myristate 13-acetate protects Jurkat cells from methylglyoxal-induced apoptosis by preventing c-Jun N-terminal kinase-mediated leakage of cytochrome c in an extracellular signal-regulated kinase-dependent manner. Mol Pharmacol.

[B53] Marriott HM, Bingle CD, Read RC, Braley KE, Kroemer G, Hellewell PG, Craig RW, Whyte MK, Dockrell DH (2005). Dynamic changes in Mcl-1 expression regulate macrophage viability or commitment to apoptosis during bacterial clearance. J Clin Invest.

